# Inhibitory Effects of Neochamaejasmin B on P-Glycoprotein in MDCK-hMDR1 Cells and Molecular Docking of NCB Binding in P-Glycoprotein

**DOI:** 10.3390/molecules20022931

**Published:** 2015-02-11

**Authors:** Lanying Pan, Haihong Hu, Xiangjun Wang, Lushan Yu, Huidi Jiang, Jianzhong Chen, Yan Lou, Su Zeng

**Affiliations:** 1 Laboratory of Pharmaceutical Analysis and Drug Metabolism, Zhejiang Province Key Laboratory of Anti-Cancer Drug Research, College of Pharmaceutical Sciences, Zhejiang University, Hangzhou 310058, China; E-Mails: lanyingpan@126.com (L.P.); haihongh2006@163.com (H.H.); yuls@zju.edu.cn (L.Y.); hdjiang@zju.edu.cn (H.J.); chjz@zju.edu.cn (J.C.); 2 Laboratory of Natural Medicine, School of Forestry and Bio-technology, Zhejiang A&F University, Lin’an 311300, China; E-Mail: wangxjchina@126.com; 3 The First Affiliated hospital, College of Medicine, Zhejiang University, 79 Qingchun Road, Hangzhou 310000, China

**Keywords:** neochamaejasmin B, P-gp, inhibition, biflavonoid, molecular simulation, suppression

## Abstract

*Stellera chamaejasme* L. (Thymelaeaceae) is widely distributed in Mongolia, Tibet and the northern parts of China. Its roots are commonly used as “Langdu”, which is embodied in the Pharmacopoeia of the P.R. China (2010) as a toxic Traditional Chinese Medicine. It is claimed to have antivirus, antitumor and antibacterial properties in China and other Asian countries. Studies were carried out to characterize the inhibition of neochamaejasmin B (NCB) on P-glycoprotein (P-gp, ABCB1, MDR1). Rhodamine-123 (R-123) transport and accumulation studies were performed in MDCK-hMDR1 cells. ABCB1 (MDR1) mRNA gene expression and P-gp protein expression were analyzed. Binding selectivity studies based on molecular docking were explored. R-123 transport and accumulation studies in MDCK-hMDR1 cells indicated that NCB inhibited the P-gp-mediated efflux in a concentration-dependent manner. RT-PCR and Western blot demonstrated that the P-gp expression was suppressed by NCB. To investigate the inhibition type of NCB on P-gp, *K_i_* and *K_i_*’ values were determined by double-reciprocal plots in R-123 accumulation studies. Since *K_i_* was greater than *K_i_’*, the inhibition of NCB on P-gp was likely a mixed type of competitive and non-competitive inhibition. The results were confirmed by molecular docking in our current work. The docking data indicated that NCB had higher affinity to P-gp than to Lig1 ((*S*)-5,7-dihydroxy-2-(4-hydroxyphenyl)chroman-4-one).

## 1. Introduction

**Figure 1:**
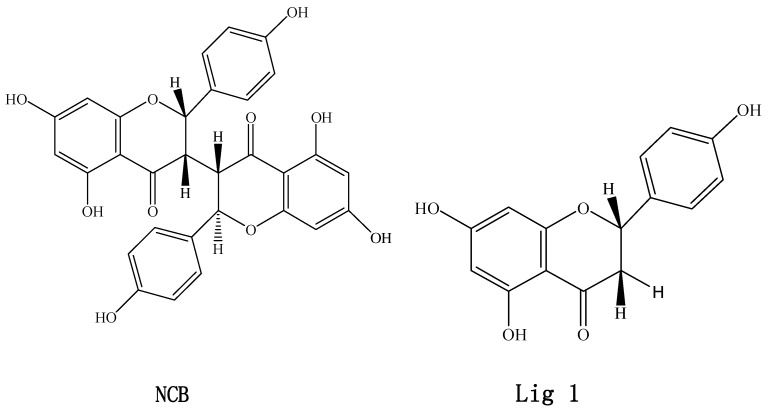
Chemical structures of neochamaejasmin B (NCB) and Lig 1 ((*S*)-5,7-dihydroxy-2-(4-hydroxyphenyl)chroman-4-one). Neochamaejasmin B was isolated and purified from the dried roots of *Stellera chamaejasme* L in our laboratory. Lig1, a single flavonoid structure corresponding to NCB, was prepared by modifying the structure of NCB using the Sketch module in SYBYL-X 1.3 (Tripos(DE), Inc., St. Louis, MI, USA).

*Stellera chamaejasme* L. (Thymelaeaceae) is a toxic perennial herb that is widespread in Mongolia, Tibet and the northern parts of China and claimed to have antivirus [1,2], antitumor [3] and antibacterial [4] properties. It is also suggested to have immune modulating activities [5] and insecticidal activity [6]. Its root, Langdu, is denoted as a toxic traditional medicine in the Pharmacopoeia of the People’s Republic of China [7]. Biflavonoids are found as the main functional constituents in this toxic herb. Neochamaejasmin B (NCB) (Figure 1) is one of the major active biflavonoids, which has the major antimicrobial activity [8]. Our previous studies have found that the oral bioavailability of chamaechromone (another biflavonoid of Langdu) was low [9] and revealed that this biflavonoid could undergo extensive phase I and phase II metabolism [10]. Besides that, to our knowledge, few previous studies have reported the absorption and metabolism of biflavonoids isolated from *Stellera chamaejasme* L.

P-glycoprotein (also called ABCB1), an important member of the ATP-binding cassette [11] transporter family, is the main efflux pump with a broad substrate spectrum. P-gp is the critical efflux transporter with dominant expression in most excretory and barrier-function tissues. Although substantial evidence has demonstrated the important role of P-gp in the absorption and distribution of flavonoids [12,13,14], only a few reports are available for bioflavonoids [15]. Thus, it is crucial to understand the transport mechanism mediated by ABC pumps. In the present study, the effects of NCB on the function and expression of P-gp were investigated in MDCK-hMDR1 cells for the first time. A detailed characterization of its inhibition mechanism is discussed.

## 2. Results and Discussion

### 2.1. Cytotoxicity of NCB for in Vitro Incubation

After cells were incubated with NCB (up to 156.25 μM) for 3 h, there was no toxicity observed. However, the cytotoxicity of NCB was detected after incubation for 40 h. Its IC_50_ was 20.60 μmol·L^−1^ for MDCK and 210.9 μmol·L^−1^ for MDCK-hMDR1 cells by the MTT assay, respectively (*n* = 3, 4). The study indicated that the cytotoxicity of NCB at a concentration 50.0 μM was minimal, and it was suitable for subsequent transporter experiments.

### 2.2. R-123 Transport Studies

**Figure 2:**
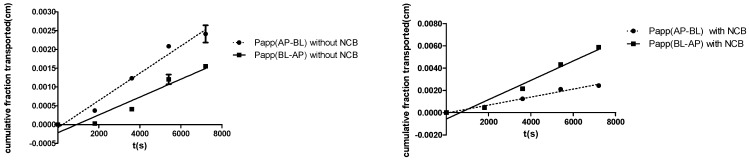
Polarized efflux of R-123 in MDCK-hMDR1 was shown to be inhibited by adding NCB (50 μM) to the mixture during 2.5 h of incubation. R-123 was performed. Apparent permeability (P_app_; BL-AP; BL, basolateral; AP, apical) with NCB, P_app_ (AP-BL) with NCB, P_app_ (AP-BL) without NCB and P_app_ (AP-BL) without NCB determined the secretory (BL-AP) transport direction of R-123 in the presence of NCB and in the absence of NCB, respectively. P_app_ (AP-BL) without NCB refers to the cell monolayers incubated in Hank’s Balanced Salt Solution (HBSS). The symbols show the data points, with error bars showing the standard deviation for six independent monolayers.

To evaluate whether NCB inhibited the P-gp-mediated efflux in MDCK-hMDR1 cells, Rhodamine-123 (R-123), a known substrate of P-gp, was selected. When co-incubated with NCB (50 μM), the efflux ratio (ER) of R-123 decreased from 6.59 ± 0.14 to 2.41 ± 0.062, and the percentage was decreased to 36.6%, which indicated that NCB inhibited R-123 efflux mediated by P-gp (Table 1 and Figure 2). The result revealed that the apparent permeability (P_app_, cm/s) in the apical-to-basolateral direction (AP-BL) of R-123 was similar to that observed in the absence or presence of NCB, and the P_app_ in the basolateral-to-apical direction decreased significantly when exposed to NCB (Figure 2). Thus, polarized efflux of R-123 in MDCK-hMDR1 cells was observed, and the inhibition of P-gp-mediated R-123 efflux was found by adding NCB (50 μM) to the incubation mixture. The inhibition was confirmed by the following studies.

**Table 1:** Effects of NCB on the transport of R-123 across MDCK-hMDR1 cell monolayers.

Compound	Papp (cm/s) × 10^−7^		Efflux Ratio (P_app BL-AP_/P_app__AP-BL_)
	BL-AP	AP-BL	
R-123	23.9 ± 1.54	3.63 ± 0.31	6.59 ± 0.14
R-123 + 50 μM NCB	8.67 ± 1.02	3.59 ± 0.33	2.41 ± 0.062

Data are shown as the mean ± SD for six independent monolayers. *p* < 0.05.

### 2.3. R-123 Accumulation Assay

**Figure 3:**
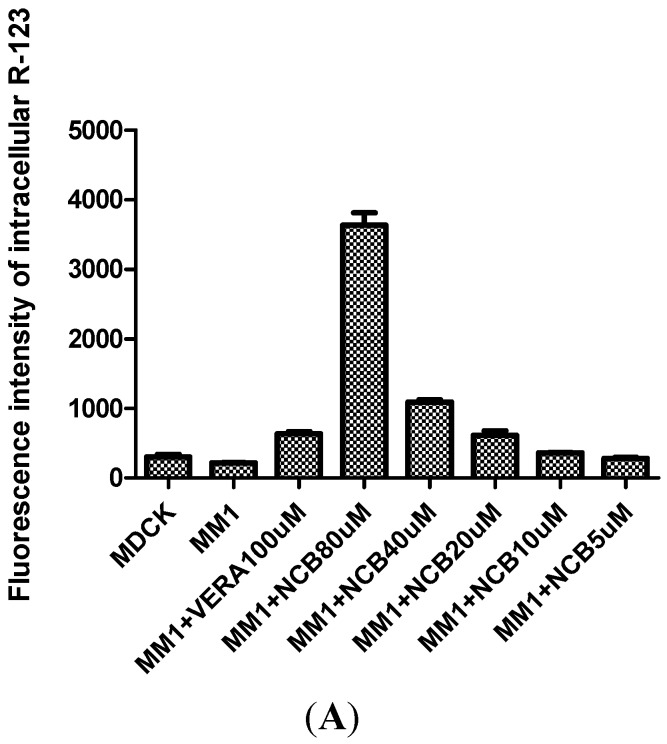

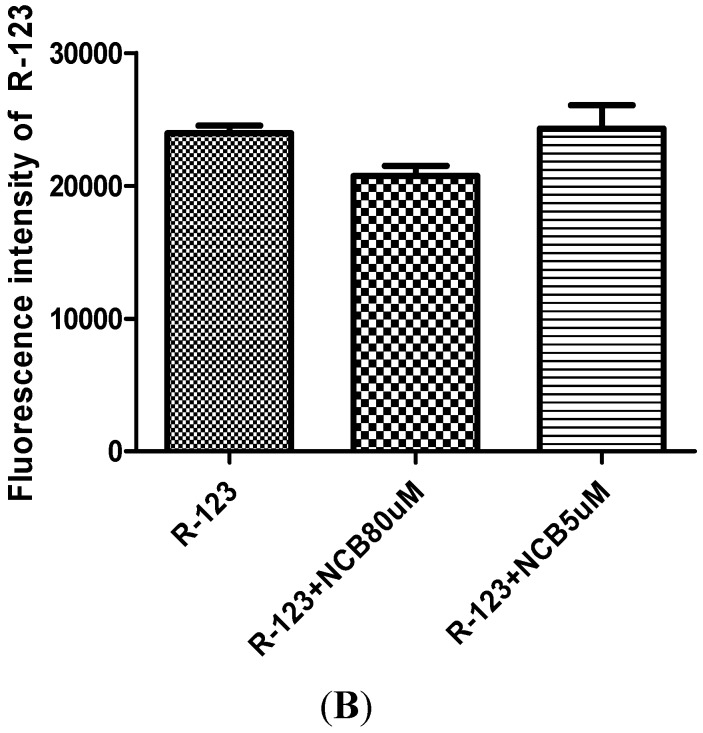
R-123 accumulation assays in MDCK and MDCK-hMDR1 Cells. For co-incubation with different concentrations of NCB and verapamil, the R-123 accumulation was dose-dependent on NCB (**A**) (MDCK denotes MDCK cells; MM1 denotes MDCK-hMDR1 cells). Fluorescence assays of R-123 co-incubation with different concentrations of NCB (**B**). No fluorescence quenching or enhancement of R-123 was observed when different concentrations of NCB were added.

R-123 accumulation in MDCK-hMDR1 cells indicated that P-gp was stably overexpressed [16]. After co-incubated with NCB (80 μM), the intracellular accumulation of R-123 was 5.7-fold higher than verapamil (100 μM), a P-gp inhibitor [17]. The results indicated that NCB was an inhibitor. Notably, as shown in Figure 3A, R-123 accumulation was dose-dependent with NCB. As Figure 3B shows, no fluorescence quenching and enhancement of R-123 were observed.

### 2.4. K_i_ and K_i_’ Assay

Inhibitory constants *K_i_* and *K_i_*’ are important parameters in the kinetics of transport inhibition. They determine the types of NCB inhibition. To investigate the inhibition type of NCB interactions with P-gp, inhibitory constants *K_i_* and *K_i_*’ were further studied.

**Figure 4:**
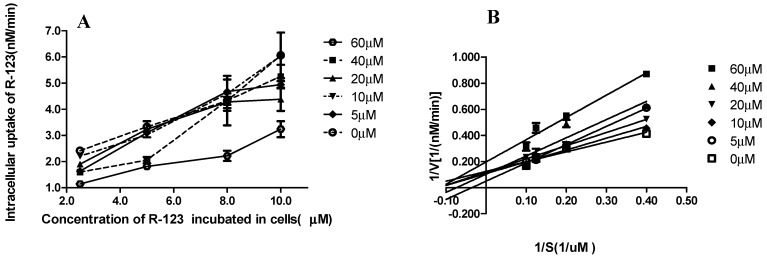

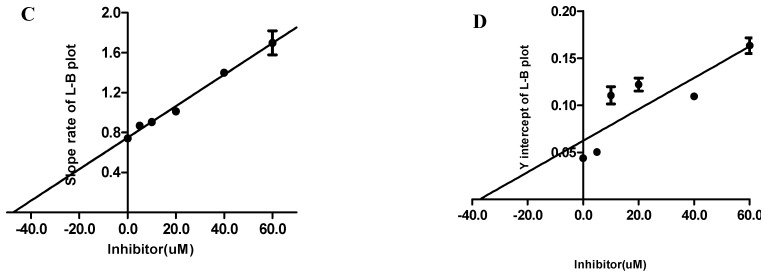
Lineweaver-Burk plots. Intracellular uptake velocity of R-123 (nM/min) was examined in cells incubated with R-123 (**A**). Cells were incubated in different concentrations of NCB (0, 5.0, 10, 20, 40, 60 μM) at 37 °C, for 60 min. The concentrations of R-123 were 0, 5.0, 8.0, 10.0 μM. The Lineweaver-Burk plots were obtained by plotting 1/*v* as the ordinate against 1/[S] as the abscissa (**B**). The data were used to calculate the *K_i_* and *K_i_*’ values of NCB on the active accumulation of R-123 in MDCK-hMDR1 cells. Reciprocals of intracellular R-123 (0, 5.0, 8.0, 10.0 μM) in MDCK-hMDR1 cell content velocity *vs*. the reciprocals of different concentrations of NCB (0, 5.0, 10, 20, 40, 60 μM) at 37 °C, for 60 min, were calculated. Data points are the mean ± SD (*n* = 3). The *K_i_* value for P-gp-mediated NCB was calculated (**C**). The slope rate of the Lineweaver-Burk plot *vs.* different concentrations of NCB (0, 5.0, 10, 20, 40, 60 μM) is shown. Using these data, the *K_i_* value for NCB was calculated using the equation as described in the Materials and Methods. Data points were the mean ± SD (*n* = 3). The *K*_i_’ value for P-gp-mediated NCB was calculated (**D**). The Y-axis intercept of the Lineweaver-Burk plot *vs.* different concentrations of NCB (0, 5.0, 10, 20, 40, 60 μM) was shown. Using these data, the *K_i_*’ value for NCB was calculated using the equation as described in Materials and Methods. Data points were the mean ± SD (*n* = 3).

The protein concentration of the transporter determined by the BCA kit protein assay was 0.27 μg·mL^−1^. According to the inhibitory reaction rate equation, *K*_i_ and *K*_i_’ for NCB inhibition of P-gp can be obtained by double-reciprocal plots. A Lineweaver-Burk plot was obtained by plotting 1/*v* as the ordinate against 1/[S] as the abscissa (Figure 4A,B). Additionally, for quadratic mapping, *K*_i_ was calculated by the slope rate of Lineweaver-Burk plot *vs.* different concentrations of NCB (0, 5.0, 10, 20, 40, 60 μM). *K*_i_’ was calculated by the Y-axis intercept of the Lineweaver-Burk plot *vs.* different concentrations of NCB (0, 5.0, 10, 20, 40, 60 μM) (*n* = 3). According to the data, *K*_i_ was 45.25 μM and *K*_i_’ was 39.45 μM (Figure 4C,D). Since *K*_i_ was greater than *K*_i_’, this suggested that the inhibition effect of NCB on P-gp was the mixed type of competitive and non-competitive inhibition. 

### 2.5. Quantitative Determination of ABCB1 mRNA Expression

**Figure 5:**
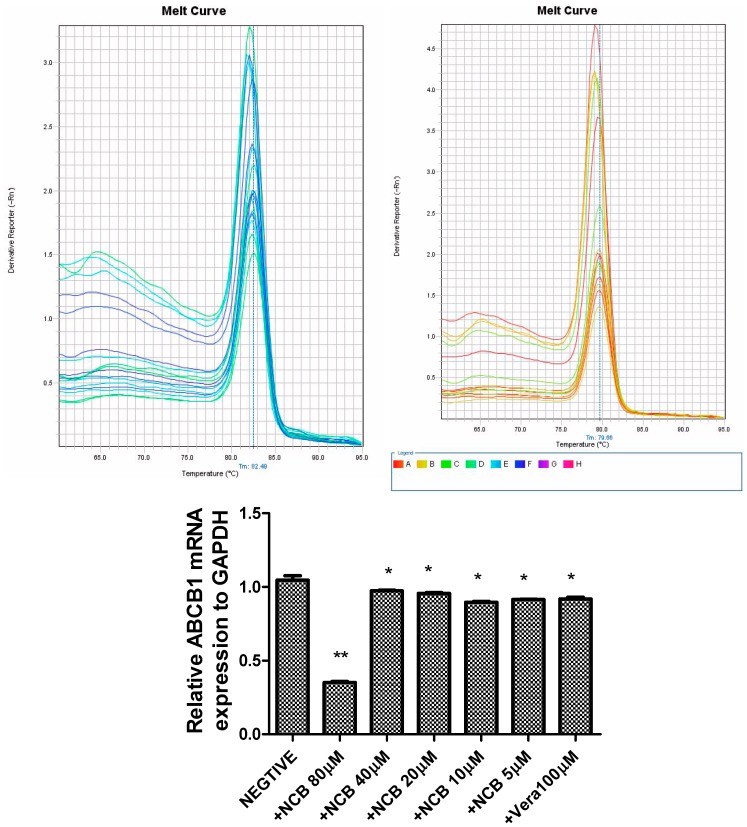
ABCB1 mRNA expression in the presence or absence of NCB and verapamil. ABCB1 mRNA was quantified by RT-PCR in MDCK-hMDR1 cells. Additionally, the relative gene expression levels were analyzed by the 2^−ΔCt^ method. Negative, +NCB 80 μM, +NCB 40 μM, +NCB 20 μM, +NCB 10 μM, +NCB 5 μM and +Vera 100 μM refer to cells treated with HBSS, 80 μM NCB, 40 μM NCB, 20 μM NCB, 10 μM NCB, 5 μM NCB and 100 μM verapamil (figure above: Melting curve of GAPDH and ABCB1). * Statistically significant difference compared with controls (*p* < 0.05), ** Statistically significant difference compared with controls (*p* < 0.01).

ABCB1 mRNA was quantified using a quantitative RT-PCR assay in MDCK-hMDR1 cells co-incubated with NCB over 24 h. Relative gene expression levels were analyzed by the 2^−ΔCt^ method. After cells were incubated with 80 μM NCB, ABCB1 mRNA expression decreased to one-third compared to cells incubated with HBSS (*p* < 0.001). When the cells were incubated with NCB (5–40 μM), ABCB1 mRNA expression decreased to ninety percent compared to cells incubated with HBSS (*p* < 0.05). As a result, the inhibition of NCB to P-gp in MDCK-hMDR1 cells showed that the P-gp expression was suppressed by NCB over time (Figure 5). However, a dose correlation was not observed.

### 2.6. Western Blot Analysis

**Figure 6:**
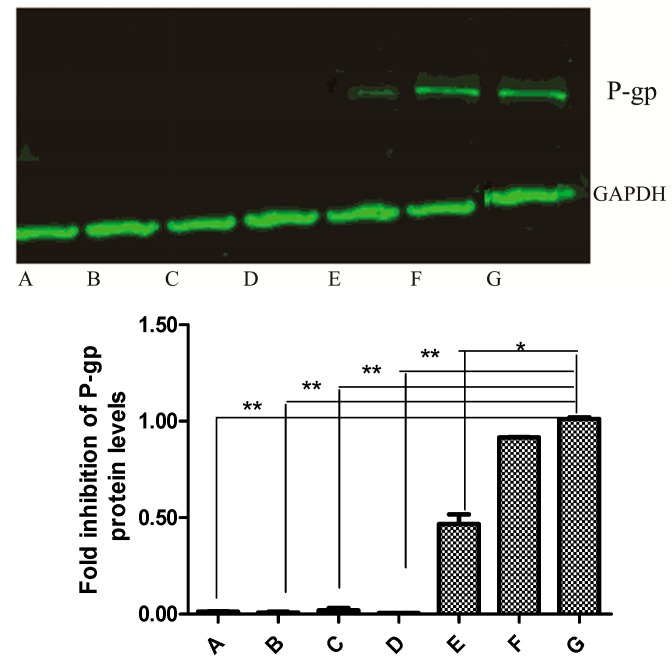
Effect of NCB on P-gp protein expression in MDCK and MDCK-hMDR1 cells. Whole-cell extracts were analyzed by Western blot with a specific anti-P-gp antibody. The upper arrow indicates P-gp protein, and the lower one indicates the endogenous GAPDH protein. A, B, C, D, E, F and G refer to different protein samples: A, MDCK cells incubated with HBSS (negative control); B, MDCK-hMDR1 cells incubated with NCB (80 μM); C, MDCK-hMDR1 cells incubated with NCB (40 μM); D, MDCK-hMDR1 cells incubated with NCB (20 μM); E, MDCK-hMDR1 cells incubated with NCB (10 μM); F, MDCK-hMDR1 cells incubated with Vera (100 μM); G, MDCK-hMDR1 cells incubated with HBSS (positive control). * Statistically significant difference compared with controls (*p* < 0.05), ** Statistically significant difference compared with controls (*p* < 0.01).

Western blot analysis showed a decrease in P-gp protein level after MDCK-hMDR1 cells were exposed to 10 μM NCB for 24 h. Additionally, the protein fluorescence band faded away upon exposure to NCB (20-80 μM), which suggested that NCB resulted in the downregulation of P-gp expression without dose-dependent correlation. Consequently, this suggested that the P-gp expression was suppressed by NCB over time (Figure 6).

### 2.7. Binding Selectivity Studies Based on Molecular Docking

**Figure 7:**
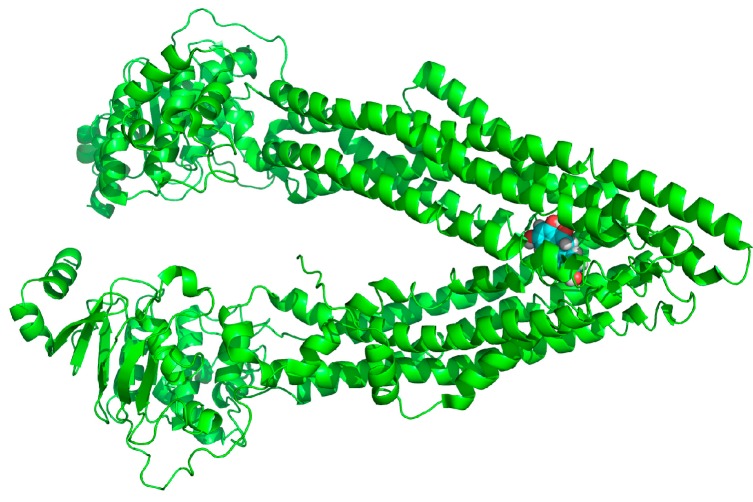
The structure of P-gp (green) and a pocket snapshot of NCB (cyan and white) from the molecular docking model.

**Figure 8:**
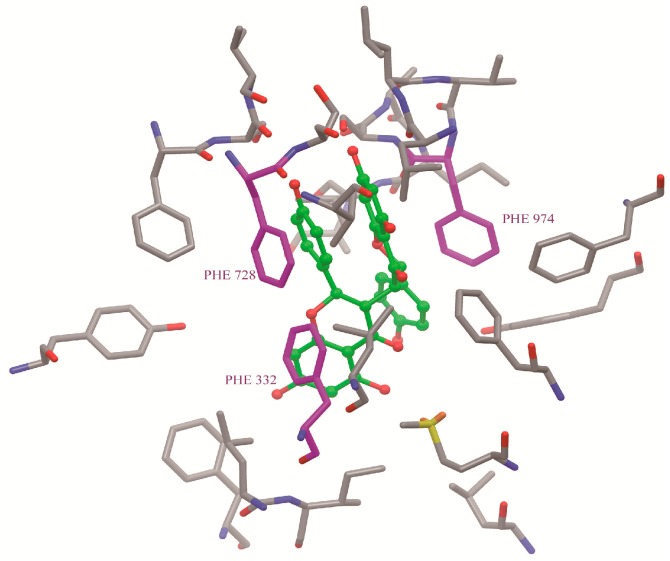
The binding site (yellow) of NCB (cyan) to P-gp was analyzed by docking simulation. NCB, with a higher number of hydrophobic features and hydrogen bond acceptors, was more prone to P-gp binding. Moreover, the binding site of NCB to P-gp lies beside Phe332, Phe728 and Phe 974 in chain A. The benzene ring of NCB could also have a parallel π-π interaction with the benzene ring of P-gp. The rotations of all single bonds of the ligand and the side chains of residues within 5 Å of the ligand were considered.

**Figure 9:**
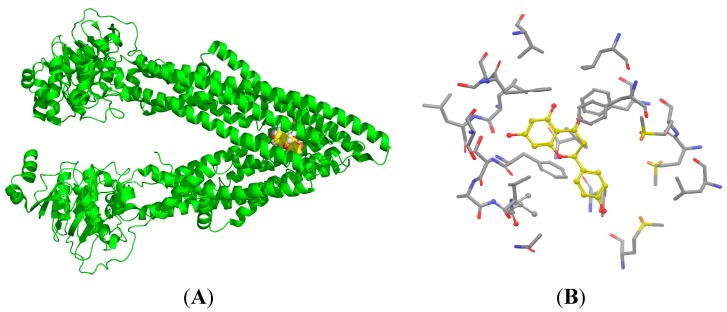
The structure of P-gp (green) and the pocket snapshot of Lig1 (yellow and white) from the molecular docking model (**A**). Additionally, the pocket snapshot is in sticks **(B**). The hydrophobic residues contributed primarily to Lig1 binding to P-gp. The rotations of all single bonds of the ligand and the side chains of residues within 5 Å of the ligand were considered.

The binding affinities of Lig1 (Figure 1) and NCB [18] predicted on the basis of the FlexiDock protocol are −72.2 and −98.0 kcal/mol, respectively. This suggested that NCB forms stronger interactions with P-gp than Lig1. Therefore, we speculated that NCB was a better inhibitor of P-gp-mediated cellular efflux compared with (Figure 7 and Figure 8) another flavonoid, Lig1 (Figure 9). 

### 2.8. Discussion

There is some increasing evidence indicating that the use of herbal products could result in clinically important drug interactions. *Stellera chamaejasme* L. (Thymelaeaceae) is one of the toxic Chinese herbs. The root is used as “Langdu” in traditional Chinese medicine. It displays therapeutic effects on some diseases, such as stubborn skin ulcers, leucocythemia and stomach cancer [19]. NCB is the major antimicrobial active ingredient in the toxic herb. Until now, this is the only study that has provided insight into the role and mechanism of biflavonoid isolated from Langdu on the transporter. Additionally, it has evidenced that flavonoids can be a natural source of P-gp inhibitors [20]. Interestingly, our study showed that NCB was not only an inhibitor, but also a substrate for P-gp. These results imply that P-gp might limit the oral bioavailability of NCB by pumping it out of the cells. A detailed characterization of its inhibition mechanisms is elucidated in the present study. According to the data, a mixed competitive and noncompetitive inhibition was implicated. Mixed inhibition is a type of inhibition in which the inhibitor may bind to the target biopolymer independent of whether or not the target biopolymer has already bound the substrate [21,22]. In mixed inhibition, the inhibitor binds to an allosteric site, *i.e*., a site different from the active site where the substrate binds [23]. The study suggested that NCB not only inhibited P-gp-mediated R-123 efflux in the initial 2.5 h, but also suppressed P-gp expression over the course of time 24 h later. This suggested that NCB promoted an active conformation in the other monomer, once bound to one site in the P-gp. Steady-state kinetic modeling supported this model [24]. However, we could not rule out the possibility of the result of tumor cell apoptosis.

P-gp is an approximately 170-kDa protein, consisting of 1,280 amino acid residues. This transmembrane single polypeptide is structurally composed of two homologous parts. Each homolog contains six trans-membrane (TM) segments followed by a consensus nucleotide-binding domain (NBD) [25]. The TM segments of P-gp are believed to be the putative sites for substrate recognition, and the NBDs are the sites for ATP binding/hydrolysis [26]. Flavonoids are capable of binding with both the ATP-binding site [27] and the vicinal steroid-binding hydrophobic region (SBHR) within the cytosolic domain of P-gp [28]. The structure-activity relationship analysis of flavonoids suggested a 2,3-double bond in the C ring [29], the 3,5-OH groups [30] and a suitable log δ, c × log P_oct_ value, which are the favorable factors for being P-gp inhibitor [31]. The increase of R-123 accumulation by nearly 20 μM NCB in MDCK-hMDR1 cells is comparable with that of 100 μM of verapamil, indicating that NCB is a fairly potent P-gp inhibitor, even though having no 2,3-double bond in the C ring. To address these issues, we carried out docking simulations of NCB on a P-gp protein model and binding free energy calculations (Figure 7). The results indicated that biflavonoids with a higher number of hydrophobic features and hydrogen bond acceptors were more prone to P-gp binding (Figure 8). Moreover, the binding site of NCB to P-gp lies beside Phe332, Phe728 and Phe 974 in chain A (Figure 8).

P-gp is upregulated in many cancer cells, where it reduces the intracellular concentrations of many chemotherapeutic drugs, thereby imparting multidrug resistance (MDR) [32]. It was reported that biflavonoid in the extracts of *Stellera chamaejasme.* L had antitumor activity [33]. The extracts induced tumor cell apoptosis, reconstruct turn signals in the apoptosis of tumor cells [34] and dramatically raise the expression levels of Fas/Fas-L and TNF-α [35], which was often used as a prognostic indicator in tumor patients after chemotherapy [34,36,37]. Our study showed that NCB suppressed ABCB1 mRNA and protein expression in the P-gp overexpressed cell line. The mechanism of reversing P-gp-mediated multi-drug resistance of NCB adds a new mechanism to its antitumor activity in addition to the mechanism by raising the expression levels of Fas/Fas-L and TNF-α.

In summary, our findings provide initial evidence that NCB can inhibit P-gp-mediated cellular efflux. The underlying mechanism(s) may involve both competitive and non-competitive inhibition. In addition, the mechanism of reversing P-gp-mediated multi-drug resistance of NCB adds a new mechanism to Langdu’s antitumor activity, a further theoretical support of MDR reversion by *Stellera chamaejasme* L.

## 3. Experimental Section

### 3.1. Materials

Neochamaejasmin B (>98.0%) (Figure 1) was isolated and purified from the dried roots of *S. chamaejasme* in our laboratory, and the structure was confirmed by MS, ^13^C- and ^1^H-NMR, as described in the literature [38]. Verapamil (Vera), cyclosporin A (CsA), R-123, dimethylsulfoxide (DMSO) and 3-(4,5-dimethylthiazolyl-2)-2,5- diphenyltetrazolium bromide (MTT) were purchased from Sigma Chemical Corporation (Saint Louis, MO, USA). Tris, glycerin and sodium dodecyl sulfate (SDS) were purchased from Bio-Rad Laboratories (Hercules, CA, USA). Dulbecco’s modified Eagle’s medium (DMEM, high-glucose), fetal bovine serum, nonessential amino acids, 0.25%trypsin-EDTA solution and antibiotic-antimycotic were purchased from Gibco BRL Life Technology (Grand Island, NY, USA). MDCK-hMDR1 cells originated from the transfection of Madin Darby canine kidney (MDCK) cells with the human ABCB1 gene in our laboratory [16]. Cell culture flasks and Transwell^®^ polycarbonate inserts (12 mm diameter, 0.4 μm pore size) were obtained from Corning Costar Corporation (Bedford, MA, USA). The AxyPrep Multisource Total RNA Miniprep Kit was purchased from Axygen Corporation (Union City, CA, USA). RNasin and reverse transcriptase, the real-time qPCR Kit and SYBR Premix Ex TaqTM were purchased from Takara (DRR036A, DRR420A, Shiga, Japan). Mouse-anti-P-gp and mouse-anti-GAPDH were purchased from Abcam (Ab10333, Cambridge, UK) and Kangche Bio-technology (Shanghai, China), respectively. The secondary antibodies were goat-anti-mouse of IRDye 700CW (Oddssey, Lincoln, NE, USA). All solvents used were HPLC grade, and all chemicals were analytical grade.

### 3.2. Cell Culture

MDCK and MDCK-hMDR1 cells were grown in DMEM with 10% FBS, 100 U/mL antibiotic-antimycotic in a humidified atmosphere of 5% CO_2_ at 37 °C. Cells were seeded in 6-well, 12-well or 48-well plates or 12-well Transwell^®^ plates. Culture medium was changed every other day.

### 3.3. In Vitro Cytotoxicity Assays

Cell viability was measured by the MTT assay. Briefly, cells were seeded in 96-well plates and treated with NCB as indicated [39,40]. After cells were incubated for 3 or 40 h, MTT (1 mg/mL) was added to the cells, which was reduced to purple formazan crystals by the mitochondria of living cells. The reduced crystals were solubilized with DMSO, and the absorbance was measured at 490 nm by a spectrophotometer (Bekman Corporation, Fullerton, CA, USA).

### 3.4. R-123 Transport Experiments

Bidirectional transport experiments were performed. All experiments were done at 37 °C in air with constant mixing in a shaking water bath (60 rpm). Briefly, both the apical (AP) and the basolateral (BL) chambers of each insert were washed twice with 37 °C HBSS for 15 min. R-123(5 μM) as a probe substrate transported in the presence of NCB (50 μM) or verapamil (100 μM) as inhibitors was performed, too [41]. At the times of 1800, 3600, 5400 and 9000 s, a 100-μL aliquot was withdrawn from the receiver chamber and immediately replenished with an equal volume of pre-warmed HBSS. The fluorescence of R-123 was measured with microplate reader analysis at 485 nm (excitation) and 538 nm (emission). The calibration curve of R-123 was linear over the concentration range of 0.244–1000 nM [42,43].

### 3.5. Transport Experiments Data Analyses

The transport rates (V), apparent passive permeability [44] and efflux ratio were calculated as:

V = dQ/dt·A

P_app_ = dQ/dt·A·C_0_

Efflux ratio = P_app_/P_app_ (A-B)

where dQ/dt (μmol/s) is the efflux rate, A (cm^2^) is the effective surface area of the cell monolayer (1.13 cm^2^) and C_0_ (μM) is the initial drug concentration in the donor chamber. P_app_ [45] refers to P_app_ (BL-AP) and P_app_ (A-B) refers to P_app_ (AP-BL). The efflux ratio is expressed as the quotient of P_app_ (BL-AP) to P_app_ (AP-BL) [45].

Data are expressed as the mean ± SD of six determinations. The statistical software package SAS (v8.2; SAS Institute, Inc., Cary, NC, USA) was used for data analysis.

### 3.6. Cellular Accumulation

#### 3.6.1. R-123 Accumulation Assay

Intracellular uptake of R-123 as a probe substrate for P-gp was performed in MDCK-hMDR1 cells. Before initiating the uptake experiments, cells were washed three times with HBSS at 37 °C. The cells were then pre-incubated with HBSS, or 100 μM verapamil, or different concentrations of NCB for 20 min. The final concentrations of R-123 were 2.5, 5, 8 and 10 μM [46]. Cells were incubated with R-123 in the presence of inhibitor for 60 min at 37 °C. This experiment was terminated by ice-cold PBS three times. Then, the cells were lysed with 0.1% (v/v) Triton X-100 in PBS for 15 min at 37 °C. The fluorescence of R-123 in cell lysates was measured at 485 nm (excitation wavelength) and 538 nm (emission wavelength).

#### 3.6.2. R-123 Accumulation Data Analyses

##### *K_i_* and *K_i_*’ Assay

*K_i_* and *K_i_*’ were analyzed by the R-123 accumulation assay [47]. The protein concentration of the transporter was determined by the BCA protein assay. NCB concentrations were 0, 5, 10, 20, 40 and 60 μM, and R-123 concentrations were 2.5, 5, 8 and 10 μM. The time of cell accumulation was 60 min, and the *V* value was characterized as nM·min^−1^. The following equation was used for data analysis:

$$\frac{1}{V}=\frac{K_{m}}{V_{m}}\times (1+\frac{\left[I\right]}{K_{i}})\times \frac{1}{\left[S\right]}+\frac{1}{V_{m}}\times (1+\frac{\left[I\right]}{K_{i}^{\prime }})$$

Here, kinetic parameter *V_m_* is the maximum rate achieved by the system, at maximum (saturating) substrate concentrations. The Michaelis constant *K_m_* is the substrate concentration at which the reaction rate is half of *V_m_* and [S] is the concentration of the substrate.

### 3.7. Determination of ABCB1 mRNA Gene Expression

Total RNA was obtained from MDCK and MDCK-hMDR1 cells using the AxyPrep Multisource Total RNA Miniprep Kit and quantified by multiscan spectrum. Total RNA (0.5 μg) from each sample was reversed transcribed in a total volume of 10 μL mixture containing oligo d(T)15 primer, RNasin and reverse transcriptase. ABCB1 and glyceraldehyde-3-phosphate dehydrogenase (GAPDH) primers were synthesized. GAPDH was the reference gene to compare the expression of ABCB1. SYBR Green quantitative RT-PCR was conducted by a Step-One Plus q-PCR thermocycler (Applied Biosystems, Carlsbad, CA, USA). Additionally, gene expression was analyzed by the 2^−ΔCt^ method [48]. 

### 3.8. Analysis of P-gp Protein Expression by Western Blot

Total protein was electrophoresed on 8% sodium dodecyl sulfate polyacrylamide gel electrophoresis (SDS-PAGE) and blotted to the nitrocellulose (NC) membrane. After blocking with 5% skim milk in PBS for 2 h, the membranes were incubated with primary antibodies (mouse-anti-P-gp, 1:500; mouse-anti-GAPDH, 1:5000) and prepared in 3% skim milk/0.1% Tween/PBS overnight at 4 °C. The membranes were washed with 0.3% Tween/PBS 3 × 10 min and then incubated for 1 h with HRP-conjugated secondary antibodies (goat-anti-mouse IRDye 700CW, 1:15,000). Membranes were washed again and exposed to immunofluorescent reagents and then scanned with the Odyssey Infrared Imaging System. Band intensities were quantified by the Odyssey Application Software Version 3.0 [49,50]. Total protein for each cell line was determined for each run using the BCA protein assay kit, according to the manufacturer’s instructions (Pierce, Tattenhall, UK).

### 3.9. Binding Selectivity Studies Based on Molecular Docking

The co-crystal of P-gp (PDB entry: 3G5U) was obtained from the RCSB Protein Data Bank (PDB) [51]. The structures of Lig1 and NCB were constructed by the Tripos Sketch module in SYBYL-X 1.3 using the molecular fragments library [52] and relaxed by 1,000 steps of steepest descent optimizations with the Tripos force field. The optimization of the binding geometry was then carried out using the FlexiDock module in SYBYL. The rotations of all single bonds of the ligand and the side chains of residues within 5 Å of the ligand are considered. The interaction energy was evaluated using the van der Waals, electrostatic and torsional energy terms of the Tripos force field. The partial atomic charges were assigned based on the Gasteiger-Hückel formalism to evaluate the electrostatic interactions. Lig1 and NCB were manually docked into the active site of P-gp based on the geometry of the crystal structure as the template with 30 cycles of energy minimization, and the conformations of the ligands with the lowest docking scores were adopted as the binding conformation.

## 4. Conclusions

NCB inhibited the P-gp-mediated efflux in a concentration-dependent manner. The inhibition of NCB on P-gp was a mixed type of competitive and non-competitive inhibition. NCB had more appropriate selectivity to P-gp than Lig1, a single flavone structure.
